# Status of Artemisinin Resistance in Malaria Parasite* Plasmodium falciparum* from Molecular Analyses of the* Kelch13* Gene in Southwestern Nigeria

**DOI:** 10.1155/2018/2305062

**Published:** 2018-10-03

**Authors:** Mary Aigbiremo Oboh, Daouda Ndiaye, Hiasindh Ashmi Antony, Aida Sadikh Badiane, Upasana Shyamsunder Singh, Nazia Anwar Ali, Praveen Kumar Bharti, Aparup Das

**Affiliations:** ^1^Parasitology and Mycology Laboratory, Université Cheikh Anta Diop, Dakar, Senegal; ^2^Division of Vector Borne Diseases, ICMR-National Institute of Research in Tribal Health, Jabalpur, India

## Abstract

Evolution and spread of malaria parasite* Plasmodium falciparum* capable of evading antimalarials are the prime concern to malaria control. The currently effective drug, artemisinin (ART), is under threat due to detection of ART-resistant* P. falciparum* parasites in the Southeast Asian countries. It has been shown that amino acid (AA) mutations at the* P. falciparum Kelch13* (*Pfk13*) gene provide resistance to ART. Nigeria, a part of the Sub-Saharan Africa, is highly endemic to malaria, contributing quite significantly to malaria, and resistance to chloroquine (CQ) and sulfadoxine-pyrimethamine (SP) combination drugs has already been reported. Since artemisinin combined therapy (ACT) is the first-line drug for treatment of uncomplicated malaria in Nigeria and five amino acid mutations have been validated in the* Pfk13 *gene alongside with candidate mutations for ART resistance, we performed molecular surveillance for mutations (following PCR and DNA sequence analyses) in this gene from two southwestern states of Nigeria. Statistical analyses of DNA sequences were also performed following different evolutionary models. None of the different validated and candidate AA mutations of* Pfk13* gene conferring resistance to ART could be detected in* P. falciparum *sampled in the two southwestern states of Nigeria. In addition, DNA sequencing and sequence analyses indicated neither evolutionary selection pressure on the* Pfk13* gene nor association of mutations in* Pfk13* gene with mutations of other three genes conferring resistance to CQ and SP. Therefore, based on the monomorphism at the* Pfk13* gene and nonassociation of mutations of this gene with mutations in three other drug-resistant genes in malaria parasite* P. falciparum*, it can be proposed that malaria public health is not under immediate threat in southwestern Nigeria concerning ART resistance.

## 1. Introduction

Emergence and spread of drug resistance in malaria parasite,* Plasmodium falciparum*, are a severe public health concern all over the malaria endemic countries of the globe [1–3]. While the historical origin of drug-resistant* P. falciparum *goes back to Southeast Asian countries in general, in Africa, drug-resistant parasites appeared in a much later stage than in Asian countries. This reverse migration (in comparison to the evolutionary origin and spread of* P. falciparum*) has been documented for chloroquine (CQ) [4], sulfadoxine-pyrimethamine (SP) [5], and now artemisinin (ART) [6, 7]. It is now documented that ART-resistant* P. falciparum* has originated in Thai-Cambodian border [7] and dispersed to many other malaria endemic countries [8] including Africa. The* P. falciparum Kelch13* (*Pfk13*) gene encoding the propeller domain protein is located in chromosome 13 of* P. falciparum *which bears several point mutations resulting in the change of amino acid (AA), out of which only five mutations (N_458_Y, Y_493_H, R_539_T, I_543_T, and C_580_Y) have been validated to correlate with ART resistance [9, 10]. Due to these mutations,* P. falciparum *can successfully evade the only and effective drug for the treatment of uncomplicated malaria, ART. Other mutations (M_476_I, C_469_Y, C_469_F, M_476_I, K_479_I, A_481_V, R_515_K, S_522_C, and P_527_L), although present less frequently, have been associated with* in vivo*/*in vitro *resistance and few other mutations (P_553_L, F_446_I, and R_561_H) have association with delayed parasite clearance and not with* in vivo/in vitro* test; such mutations have been designated as candidate mutations of the* Pfk13* gene for ART resistance in* P. falciparum *[9]. In certain countries including Africa, no association between ART-resistant parasites and the five mutations could be found [11, 12]. This situation is a reminder of the hypothesis that drug resistance in* P. falciparum* is a highly complex mechanism, possibly involving multiple genes [11].

To this aspect, Nigeria, a part of Sub-Saharan Africa, is highly endemic to malaria, undoubtedly the highest contributor to African malaria [13]. Apart from few case reports on delayed response to ART [14, 15], and sporadic scan for AA mutations in the* Pfk13* gene, in which three nonsynonymous mutations (G_592_R, Q_613_H, and G_665_S) along with other synonymous mutations were detected [10], no systematic molecular epidemiological studies on field* P. falciparum *isolates have so far been conducted. In order to discern molecular epidemiology of* Pfk13* gene conferring resistance to ART in two southwestern states of Nigeria, we have collected field* P. falciparum *isolates to unravel (i) distributional prevalence of mutations in the part of the* Pfk13* gene containing the five validated and other candidate mutations and (ii) the possible association of mutations detected in the* Pfk13* gene with mutations in three other genes conferring resistance to CQ (*Pfcrt*) and SP (*Pfdhfr *and* Pfdhps*).

## 2. Materials and Methods

Patients with symptoms of malaria attending any of the four selected hospitals (Gbagada, Ikorodu, Akodo, and Ikate) in Lagos and two general hospitals (Central and Stella) in Edo were screened by rapid diagnostic test (RDT) kits and microscopy in the field. Blood from each patient was used to make two to three spots on Whatman® no. 3 filter paper and brought to the ICMR-National Institute for Research in Tribal Health, Jabalpur, India, after due ethical approval and transport of materials permission obtained (IRB/16/347) from Nigerian Institute of Medical Research, Yaba, Lagos state, Nigeria. In the laboratory, genomic DNA was isolated separately from a total of 436 malaria-symptomatic blood samples (using the QIAamp DNA Blood Mini Kit; Qiagen®, Hilden, Germany) and diagnostic PCR for the four malaria parasites were performed. Samples with PCR-positive results for* P. falciparum* were further processed for amplification of a DNA fragment (831 nucleotide base pair) containing the validated and candidate mutations [9, 10] of the* Pfk13 *gene using published primers [16]. Successful PCR products showing single band in gel were then subjected to PCR purification (using Fast^AP^ alkaline phosphatase and exonuclease I) and further processed for DNA sequencing by Sanger methods (an in-house facility of ICMR-NIRTH, Jabalpur) with 2X coverage (sequenced from both the forward and reverse directions). It is important to note that those isolates with multiple bands and/or those that could not be amplified were excluded from sequencing, therefore drastically reducing the number of sequenced isolates. The sequenced DNA fragments were then converted to AA sequences to identify mutations in the sequenced isolates. Using this approach, out of 151 monoinfected* P. falciparum* isolates, only 50* P. falciparum* isolates could be successfully sequenced (25 from each state) for the 831-nucleotide base pair DNA fragment of the* Pfk13* gene. DNA sequences obtained for both the Lagos and Edo state were submitted in NCBI database, with the following GenBank accession numbers: MH351212 to MH351261. For each isolate, sequence chromatograms were viewed carefully and all the sequences from a single population were aligned (with the help of BioEdit Sequence Alignment Editor v.7.0.5.3) alongside with the reference* Pfk13* sequence (GenBank accession no. Pf3D7_1343700) to detect mutations in the form of single nucleotide polymorphisms (SNPs) segregating in that particular population. Several population genetic parameters, ascertainment of SNPs, number of SNPs and haplotypes, haplotype and nucleotide diversities, and Tajima's D (TD, to test the hypothesis of neutral molecular evolution), were performed. Furthermore, to know if SNPs in different drug-resistant genes are correlated with the* Pfk13 *gene, unpublished data already generated from other three drug-resistant genes (*Pfcrt, Pfdhfr*, and* Pfdhps*) of these corresponding isolates (26 in total, where all the four sequences were available: nine from Lagos and 17 from Edo) were used to perform linkage disequilibrium (LD) between each pair of SNPs at a time.

## 3. Results and Discussion

In order to identify if mutations in the amino acids at the Kelch13 protein of* P. falciparum* associated with ART are prevalent in Nigeria, we have generated sequences of the part of the* Pfk13* gene (831-nucleotide base pair, bearing ART-resistant SNPs) from 50* P. falciparum* isolates in two southwestern Nigerian states (Lagos and Edo). We also have utilized DNA sequences of three other genes (*Pfcrt, Pfdhfr*, and* Pfdhps*) providing resistance to CQ and SP that were already generated in a different study, to gain a comprehensive understanding on the genetics of drug-resistant* P. falciparum* in southwestern Nigeria, a highly endemic country to malaria in the globe.

**Figure 1:**
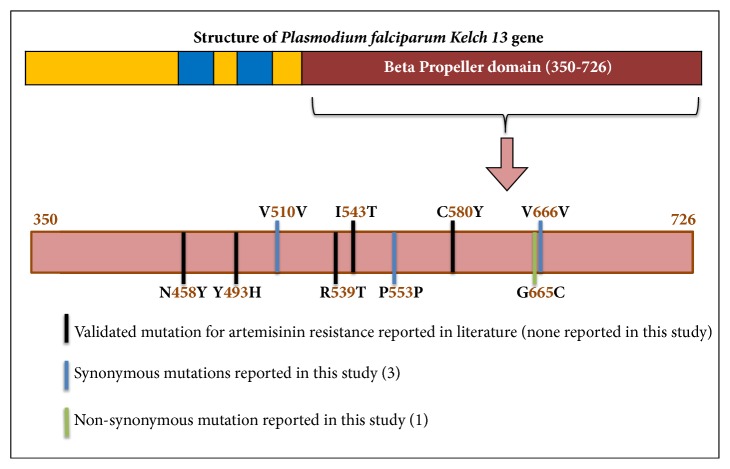
Schematic representation of the validated mutations and the mutations reported in this study in the beta propeller domain of* Kelch13* gene of* P. falciparum* in Nigerian isolates.

Interestingly, none of the five validated AA mutations and the candidate mutations associated with ART resistance [9, 10] could be detected in the 50 field isolates of* P. falciparum* indicating no evidence of ART resistance malaria parasites in southwestern Nigeria based on the present study (Figure 1). This indeed is good news for malaria epidemiology in Nigeria. ACT (artemisinin combined therapy) is the first-line drug for uncomplicated malaria in Nigeria and in the bordering countries. There is no report on the availability of either of these five validated AA substitutions or the candidate mutations in Cameroon and Benin bordering Nigeria, and also other Sub-Saharan African countries. Since African countries are aiming for malaria elimination by 2030 [13, 17], nonprevalence of mutations responsible for ART resistance is indeed a convivial news in this direction.

Apart from the validated/candidate AA mutations not detected in the present study, four synonymous mutations have been identified in the Nigerian* P. falciparum* isolates. Interestingly, P_553_P synonymous mutation was detected in Edo with a nucleotide change from CCG to CCA. This calls for a close and serious monitoring of parasites from Nigeria as the nonsynonymous AA mutation at the same 553^rd^ position changing phenylalanine to leucine (P_553_L) has taken the status of a candidate mutation for* Pfk13* marker gene [9]. Of the four mutations detected at the nucleotide level, three mutations (three haplotypes) were found to be segregated in Edo and only one (two haplotypes) was found to be segregated in Lagos. Therefore, both the haplotype and nucleotide diversities in Edo were greater (about threefold) than in Lagos (Table 1). Interestingly, the TD values from both the states were found to be negative and statistically not significantly different from neutral expectation (Table 1), indicating the* Pfk13* gene evolves under neutral model of molecular evolution. This also means that the detected point mutations at the* Pfk13* gene are not attracted by the adaptive evolution model of natural selection in* P. falciparum*. These observations with the findings on the nonavailability of the validated and candidate AA mutations responsible for ART resistance (see above) put malaria public health under control in southwestern Nigeria, at least currently.

**Figure 2:**
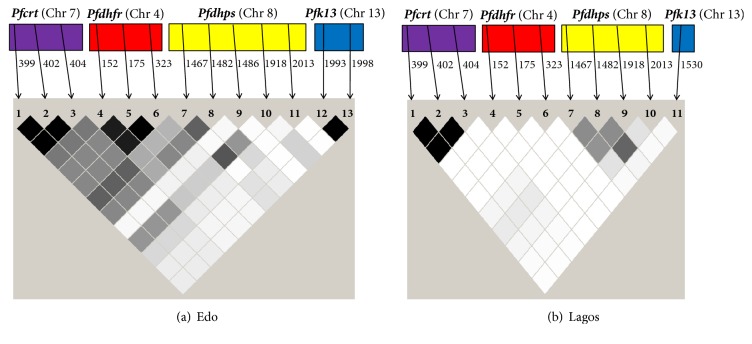
Linkage disequilibrium (LD) plots describing possible association between two different single nucleotide polymorphisms (SNPs) present either intra- or intergenetically in (a) Edo and (b) Lagos.

Several studies in the past have indicated possible associations of SNPs present in different genes conferring drug resistance (*Pfcrt, Pfdhfr, Pfdhps, Pfmdr1, etc.*) to the malaria parasite* P. falciparum* [16, 18], indicating the fact that evolution and maintenance of drug resistance in* P. falciparum* might be a product of synergistic action [19]. In order to check this hypothesis in Nigerian* P. falciparum *parasites, we utilized unpublished DNA sequence data of three other genes responsible for resistance to CQ and SP (*Pfcrt, Pfdhfr*, and* Pfdhps*) in 26 corresponding isolates (17 in Edo and 9 in Lagos) using LD analyses, and a LD plot was generated (Figures 2(a) and 2(b)). It is quite evident that significant associations between two SNPs (dark boxes) are mostly restricted to the same gene in comparison to between two different genes (grey or white boxes) in both Lagos and Edo states. However, based on the comparatively higher number of SNPs (and higher haplotype and nucleotide diversities) and haplotype and nucleotide diversities in Edo, large incidences of associations were observed (Figures 2(a) and 2(b)). Nonobservance of any statistically significant association between SNPs of two different genes, especially involving the* Pfk13* gene itself, indicates that SNPs at this gene is not in association with SNPs of any other gene conferring resistance to antimalarial in southwestern Nigeria. Although lack of association between SNPs of different genes could have been due to less number of consensus samples (17 in Edo and 9 in Lagos), utmost care has been taken in the inferential interpretation of the result to the Nigerian situation. It is therefore predicted that mutations responsible for ART resistance in the field-sampled malaria parasites from southwestern Nigeria have not appeared, perhaps due to fitness cost of survival or lack of drug pressure on circulating parasites. Likewise, mutations detected in the* Pfk13* gene in the current study did not establish any associations with mutations from other genes conferring resistance to other antimalarials. Therefore, no evolutionary selection pressure could be predicted on the existing AA mutations of the* Pfk13* gene of southwestern Nigerian* P. falciparum* isolates.

In conclusion, the present study, although restricted to (i) a limited number of field-sampled malaria parasite isolates and (ii) sampled only from the southwestern part, could enlighten on the genetic basis of ART resistance of malaria parasite,* P. falciparum*, in Nigeria. Considering ACT in different combinations (with artemisinin being the first-line drug) is the only surviving antimalarials in the control program of many endemic countries, nonobservance of validated AA mutations of* Pfk13* gene with no association among other mutations of other genes conferring resistance to partner drugs is in fact a welcome news concerning malaria public health situation in Nigeria. However, considering historical migration of CQ-resistant parasites to Africa from other different malaria endemic countries (*e.g.*, Southeast Asian countries) [20], regular and expanded molecular surveillances with large sample size on the mutations of the* Pfk13* gene are needed to monitor the prevalence of ART-resistant* P. falciparum *in Nigeria.

## Figures and Tables

**Table 1:** Genetic polymorphism and summary statistics of the *Pfk13* gene in two population samples collected in two states of southwestern Nigeria.

**State**	**Nucleotide change**	**Amino acid change**	**Number**		**Population genetic parameters**			
			**SNPs**	**Haplotypes**	**Hd**	***θ***_**w**_	***π***	**TD**
Edo (25)	**G**GT-**T**GT	G_665_C^a^	3	3	0.157	0.00096	0.00029	-1.7333
	GT**A**-GT**C**	V_666_V^b^						
	CC**G**-CC**A**	P_553_P^b^						

Lagos (25)	GT**G**-GT**A**	V_510_V^b^	1	2	0.080	0.00032	0.00010	-1.1575

^a^Nonsynonymous mutation; ^b^synonymous mutation; SNPs, single nucleotide polymorphism; Hd, haplotype diversity; *θ*_w_, number of segregating sites; *π*, average number of pairwise nucleotide differences; TD, Tajima's D.

## Data Availability

The data used to support the findings of this study are available from the corresponding author upon request.
